# The impact of obstructive sleep apnea and heart rate on arterial stiffness: results from the Tokyo Sleep Heart Study

**DOI:** 10.1038/s41440-025-02334-5

**Published:** 2025-08-22

**Authors:** Junya Kani, Kazuki Shiina, Shunichiro Orihara, Takamichi Takahashi, Hiroki Nakano, Masatsune Fujii, Megumu Saito, Chisa Matsumoto, Hirofumi Tomiyama, Kazuhiro Satomi

**Affiliations:** 1 Department of Cardiology, Tokyo Medical University, Tokyo, Japan; 2 Department of Health Data Science, Tokyo Medical University, Tokyo, Japan

**Keywords:** Obstructive sleep apnea, Heart rate, Arterial stiffness, Pulse wave velocity

## Abstract

We examined whether obstructive sleep apnea (OSA) and elevated heart rates (HR) independently increase the arterial stiffness and also the interaction between the two factors in increasing the arterial stiffness in a large Sleep Cohort. A total of 1611 subjects who underwent polysomnography and brachial-ankle pulse wave velocity (baPWV) measurement were included in the analysis. Apnea-hypopnea index (AHI) and heart rate were each categorized into three groups (non-mild: 0/h ≤ AHI < 15/h; moderate: 15/h ≤ AHI < 30/h; severe: ≥30/h; Low: HR < 70 bpm; Medium: 70 ≤ HR < 80 bpm; High: ≥80 bpm), followed by group comparisons. A significant correlation was observed between the AHI and HR. In subjects with AHI < 15, a significant increase in the baPWV was observed along with an increased HR. In subjects with HR < 70 also, a significant increase of the baPWV was observed, along with an increase of the AHI. In a crude model of mediation analysis, the AHI was found to exert a direct and indirect (via HR) effect on the baPWV. After adjustments for the age, sex, BMI, MBP, and medication status, the analysis identified AHI as showing a significant association with the baPWV mediated by the HR, whereas no significant direct relationship was observed between the AHI and the baPWV. In conclusion, in subjects with OSA, the observed increase in arterial stiffness may be mediated by an elevated HR, and therefore, elevated HR may be one of key to increase arterial stiffness in OSA.

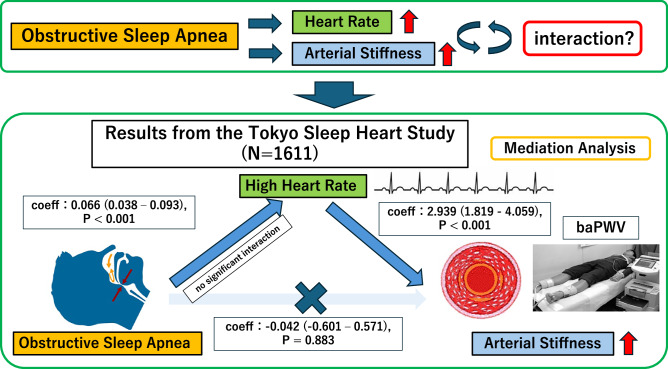

## Introduction

Both obstructive sleep apnea (OSA) and an elevated heart rate (HR) have been demonstrated as being independent risk factors for the development of cardiovascular disease (CVD) [1, 2]. Several studies have reported that both act to increase the arterial stiffness [3, 4], which is also an independent risk factor for the development of CVD [5]. Thus, while increased arterial stiffness may be involved in the increased CVD risk associated with both elevated HR and OSA, the precise mechanism by which OSA and a high HR increase arterial stiffness is not yet fully clear.

In patients with OSA, a high HR is known to be a result of increase in the sympathetic tone, and increase of the sympathetic tone is also thought to be one of key causes of increase in arterial stiffness [6]. In the general population, on the other hand, elevated HRs are known to independently increase the arterial stiffness via other mechanisms also besides sympathetic tone activation, including a direct hemodynamic pulsatile stress on the arterial wall caused by the high HR itself and inflammation/oxidative stress [7, 8]. However, it is not clear whether OSA and high HR are independently risk factors for the increase in arterial stiffness observed in patients with OSA. If the two factors acted independently to increase the arterial stiffness, they may function additively increase the arterial stiffness, which would imply that OSA and elevated HRs might serve to additively increase the risk of CVD. Furthermore, recent interventional studies have suggested that reduction of the HR leads to better CV outcomes in patients with OSA [9, 10]. Therefore, it is important to clarify whether OSA and high HRs act independently to increase the arterial stiffness and also whether the two factors interact to increase the arterial stiffness.

In this cross-sectional study, we examined whether OSA and high HR independently increase the arterial stiffness and also the interaction between the two factors in increasing the arterial stiffness in a Sleep Cohort.

Point of view

- Clinical relevance
  In a large-scale OSA cohort in Japan, the observed increase in arterial stiffness may be mediated by an elevated HR.
- Future direction
  Future research is warranted to investigate whether HR elevation in OSA patients serves as a CV risk factor through increasing the baPWV. Additionally, it is necessary to examine whether the reduction in baPWV through a decrease of the HR following initiation of CPAP might contribute to a reduction in the CV risk.
- Consideration for the Asian population
  In comparison to Western populations, OSA in Asian populations is more frequently observed in non-obese individuals. This distinct phenotype may underlie the involvement of HR in increased baPWV observed among Asian patients with OSA.

## Methods

### Patient selection

**Fig. 1:**
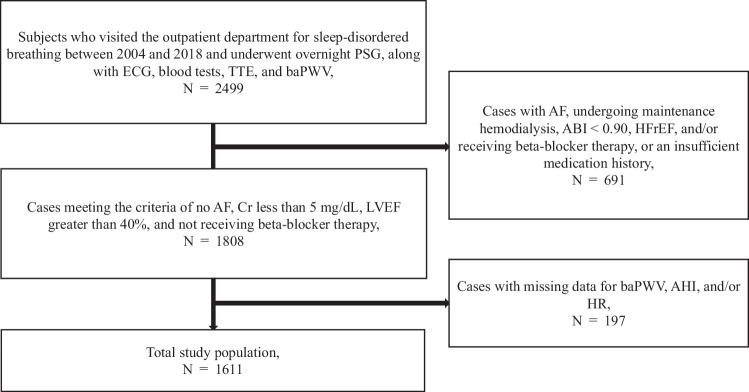
Flow-diagram of selection of the study subjects. PSG polysomnography, ECG electrocardiogram, TTE trans-thoracic echocardiography examination, baPWV brachial-ankle pulse wave velocity, AF atrial fibrillation, ABI ankle-brachial index, HFrEF heart failure with reduced ejection fraction, AHI apnea-hypopnea index, HR heart rate

Between 2004 and 2018, a total of 2499 cases underwent polysomnography (PSG) and brachial-ankle pulse wave velocity (baPWV) measurement at our Sleep Apnea outpatient clinic (Tokyo Sleep Heart Study) [11]. The study protocol for the Tokyo Sleep Heart Study has been reported elsewhere [11]. Among the 2499 subjects, 691 cases with any one or more of the following exclusion criteria were excluded from the study: unreliable accuracy of the measured baPWV values (presence of atrial fibrillation, undergoing maintenance hemodialysis, and an ankle-brachial systolic blood pressure index: ABI < 0.90); comorbidities that may affect PWV values (presence of heart failure with reduced ejection fraction: HFrEF) [12]; use of medications that affect HR (current or past history of use of beta blockers), or an insufficient medication history. In addition, 197 cases were excluded due to missing values for the baPWV, apnea-hypopnea index (AHI), or heart rate. The remaining 1611 cases were included in the analysis in the present study (Fig. 1).

### Sleep study

All the subjects underwent polysomnography (Alice 6 sleep system) in a hospital sleep laboratory. Electroencephalography, electro-oculography, electromyography, and electrocardiography were performed simultaneously and visually scored according to standard criteria [13]. Ventilatory flows at the nose and mouth were measured with thermistors. The arterial oxygen saturation was measured transcutaneously at the fingertip by pulse oximetry. Apnea was defined as a continuous cessation of airflow for more than 10 s, while hypopnea was defined as an at least 50% reduction of the airflow for more than 10 s with an oxygen desaturation of ≥3% and reduction of the chest wall movement. The apnea-hypopnea index was calculated as the total number of episodes of apnea and hypopnea per hour of sleep.

### Brachial-ankle pulse wave velocity and heart rate

The baPWV was measured using a volume-plethysmographic apparatus (Form/ABI; Colin Co.Ltd., Komaki, Japan), in accordance with a previously described methodology [14]. Briefly, electrocardiographic electrodes were placed on both wrists, and a microphone for the phonocardiogram was attached on the left chest. The electrocardiogram and phonocardiogram were used to provide timing markers for the device. Occlusion cuffs, which were connected to both plethysmographic and oscillometric sensors, were tied around both the upper arms and ankles while the participants lay in the supine position. The brachial and post-tibial arterial pressures were measured with the oscillometric sensor. The brachial and post-tibial arterial pressure waveforms determined by the plethysmographic sensor and recorded for 10 s were stored. The measurements were conducted in an air-conditioned room (24–26 °C) ear-marked exclusively for this purpose after the participants had rested for at least 5 min in the supine position. Blood pressure via the oscillometric sensor and HR via electrocardiograms were also simultaneously recorded during the measurement of the baPWV. The baPWV was measured at around 2:00 PM on the day of hospitalization for polysomnography in all cases. The HR used in the analysis was measured during baPWV measurements.

### Laboratory measurements

Fasting serum levels of high-density lipoprotein (HDL) cholesterol, low-density lipoprotein (LDL) cholesterol, triglycerides (TG), glycated hemoglobin (HbA1c), and creatinine were measured using standard enzymatic methods. All the blood samples were obtained in the morning after the patients had fasted overnight at the time of administering the sleep study.

### Statistics

Data are expressed as means ± SD. Linear associations were assessed by univariate linear regression analysis. Both the severity of sleep apnea (AHI) and the heart rate were each categorized into three groups, and one-way analysis of variance (ANOVA) and post hoc analyses (Tukey’s test) were performed for group comparisons. The direct and indirect effects of the variables on the baPWV were assessed by mediation analysis (PROCESS, ver. 3.5). All statistical tests were 2-tailed, and *P* < 0.05 was considered as denoting significance. All analyses were conducted using IBM/SPSS version 25.0 (IBM/SPSS, Chicago, IL, USA).

## Results

Table 1 presents the clinical characteristics of the 1611 patients included in the present study. The mean age was 54 ± 12 years, and the mean BMI was 26.6 ± 4.7 kg/m^2^. There were 467 smokers (29.0%), 791 patients with hypertension (49.1%), 1055 patients with dyslipidemia (65.5%), and 276 patients with type 2 diabetes (17.1%). The mean systolic blood pressure was 127.2 ± 15.2 mm Hg, the mean diastolic blood pressure was 76.2 ± 10.7 mm Hg, the serum LDL cholesterol level was 119.5 ± 30.0 mg/dl, and the serum creatinine level was 0.80 ± 0.20 mg/dl. 26.9% and 23.7% of patients were on calcium channel blockers and angiotensin II receptor blockers therapy, respectively. 19.4% of patients were on anti-dyslipidemic medications therapy.

**Table 1:** Clinical characteristics

Variables	Total	Low HR (HR < 70 bpm)	Medium HR (70 ≤ HR < 80 bpm)	High HR (HR ≥ 80 bpm)
*n*	1611	1056	358	197
Age, years	54 ± 12	55 ± 12	52 ± 12*	49 ± 12*☨
Male sex, *n* (%)	1337 (83.0)	878 (83.1)	296 (82.9)	163 (82.7)
BMI (kg/m)	26.6 ± 4.7	25.8 ± 4.2	27.5 ± 5.0*	29.3 ± 5.5*☨
SBP (mm Hg)	127.2 ± 15.2	125.5 ± 14.8	128.9 ± 15.1*	133.3 ± 16.0*☨
DBP (mm Hg)	76.2 ± 10.7	75.2 ± 10.0	78.7 ± 10.9*	80.8 ± 11.3*☨
MBP (mm Hg)	92.9 ± 11.7	91.2 ± 11.0	94.8 ± 12.2*	98.2 ± 12.2*☨
Heart rate (bpm)	66.4 ± 10.4	60.3 ± 5.6	73.6 ± 2.9*	85.9 ± 5.5*☨
AHI (*n*/h)	38.7 ± 23.3	35.4 ± 21.0	42.3 ± 24.3*	50.0 ± 28.4*☨
baPWV (cm/s)	1482.1 ± 281.9	1466.4 ± 280.0	1509.5 ± 290.9*	1516.3 ± 269.9*☨
Smoker, *n* (%)	467 (29.0)	297 (28.1)	110 (30.8)	60 (30.5)
Hypertension, *n* (%)	791 (49.1)	496 (47.0)	177 (49.6)	118 (60.0) *☨
Dyslipidemia, *n* (%)	1055 (65.5)	665 (63.0)	246 (68.9)	144 (73.1) *
Diabetes mellitus, *n* (%)	276 (17.1)	149 (14.1)	66 (18.5) *	61 (31.0) *☨
HDL-cholesterol (mg/dl)	52.0 ± 14.9	52.8 ± 15.7	50.6 ± 12.7*	50.2 ± 13.5*
LDL-cholesterol (mg/dl)	119.5 ± 30.0	119.5 ± 29.6	119.4 ± 29.7	119.4 ± 32.6
TG (mg/dl)	161.7 ± 99.2	152.5 ± 89.7	176.2 ± 115.7*	184.4 ± 108.9*
HbA1c (%)	6.0 ± 1.0	5.8 ± 0.8	6.1 ± 1.1*	6.4 ± 1.4*☨
Cr (mg/dl)	0.80 ± 0.20	0.81 ± 0.19	0.79 ± 0.22	0.78 ± 0.20
Anti-hypertensive medications				
α-blockers, *n* (%)	34 (2.1)	22 (2.1)	8 (2.2)	4 (2.0)
ACE-Is, *n* (%)	40 (2.5)	23 (2.1)	10 (2.8)	7 (3.6)
ARBs, *n* (%)	382 (23.7)	244 (23.1)	88 (24.6)	50 (25.3)
CCBs, *n* (%)	434 (26.9)	286 (27.1)	90 (25.2)	58 (29.4)
Diuretics, *n* (%)	105 (6.5)	68 (6.4)	25 (7.0)	12 (6.1)
Anti-diabetic medications, *n* (%)	132 (8.2)	73 (6.9)	36 (10.0)*	23 (11.7)*
Anti-dyslipidemic medications, *n* (%)	312 (19.4)	200 (18.9)	72 (20.2)	40 (20.3)

Values are mean ± SD or no. (%)

*HR* heart rate, *bpm* beats per minute, *BMI* body mass index, *SBP* systolic blood pressure, *DBP* diastolic blood pressure, *MBP* mean blood pressure, *AHI* apnea-hypopnea index, *baPWV* brachial-ankle pulse wave velocity, *HDL-cholesterol* serum high-density lipoprotein cholesterol, *LDL-cholesterol* serum low-density lipoprotein cholesterol, *TG* serum triglycerides, *HbA1c* serum glycated hemoglobin, *Cr* serum creatinine, *ACE-Is* angiotensin converting enzyme inhibitors, *ARBs* angiotensin II receptor blockers, *CCBs* calcium channel blockers

*p < 0.05 vs. Low HR group; ☨*p* < 0.05 vs. Medium HR group

**Fig. 2:**
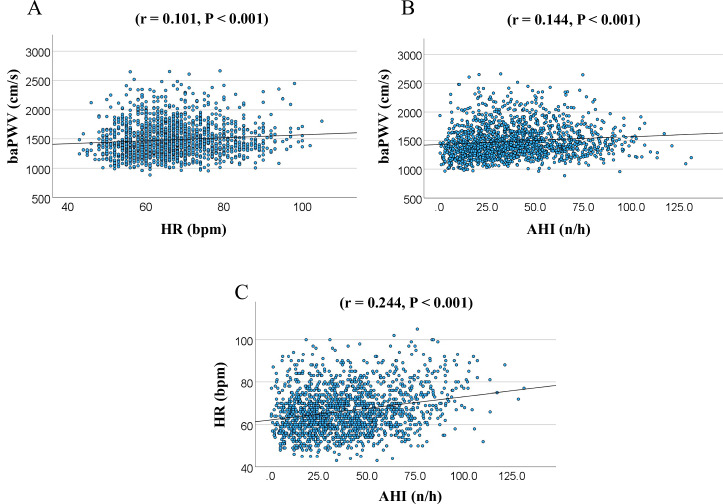
The correlation between HR, AHI, and baPWV. HR heart rate, AHI apnea-hypopnea index, baPWV brachial-ankle pulse wave velocity. **A** The correlation between HR and baPWV. **B** The correlation between AHI and baPWV. **C** The correlation between AHI and HR

The significances of the associations of the AHI and HR with the baPWV were assessed by univariate linear regression analyses. In the univariate analysis conducted without adjustments, both HR (*r* = 0.101, *P* < 0.001) and AHI (*r* = 0.144, *P* < 0.001) were identified as showing a significant correlation with the baPWV (Fig. 2A, B). In addition, the AHI also showed a significant correlation with the HR (*r* = 0.244, *P* < 0.001)(Fig. 2C). However, the correlation coefficients between baPWV and either HR or AHI were too small.

**Fig. 3:**
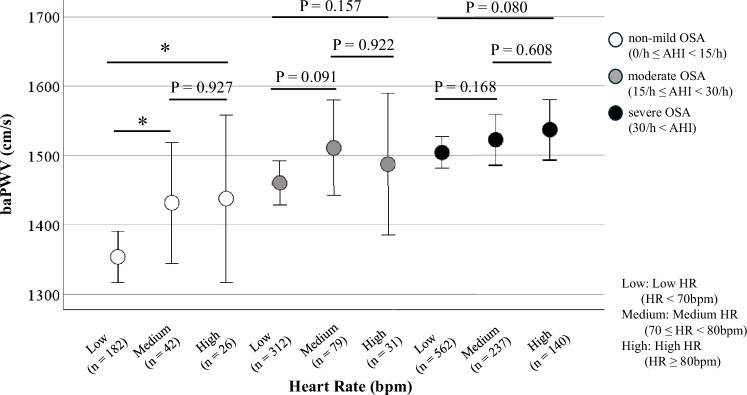
Brachial-ankle pulse wave velocity values within the heart rate value along with the severity of sleep apnea syndrome. We classified subjects with each severity level of OSA into HR value, as follows: (AHI: non-mild: (white circles): 0/h ≤ AHI < 15/h; moderate (gray circles): 15/h ≤ AHI < 30/h, and severe (black circles): AHI ≥ 30/h; HR: Low < 70 bpm; Medium: 70 ≤ HR < 80 bpm, High: HR ≥ 80 bpm), and conducted multigroup comparisons of the baPWV values. Only in cases with non-mild OSA (0/h ≤ AHI < 15/h), a significant increase of the baPWV was observed along with increase of the HR. OSA obstructive sleep apnea, AHI apnea-hypopnea index, bpm beats per minute, HR heart rate, baPWV brachial-ankle pulse wave velocity; *p < 0.05 vs. Low HR group

**Fig. 4:**
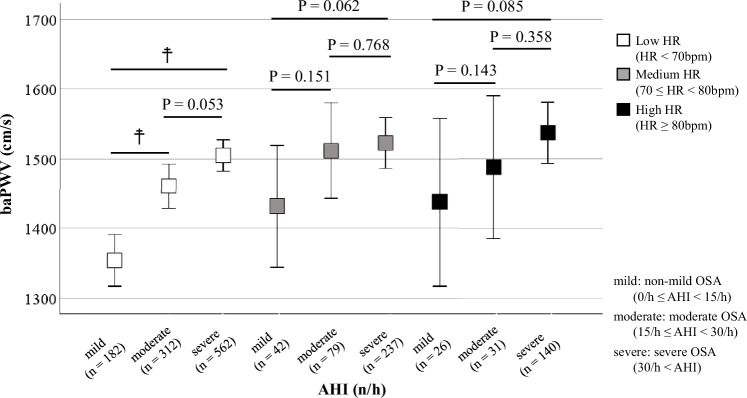
Brachial-ankle pulse wave velocity values within the severity of sleep apnea syndrome along with the heart rate value. We classified subjects in each HR value into OSA group; HR: Low (white squares): HR < 70 bpm; Medium (gray squares): 70 ≤ HR < 80 bpm; High (black squares) ≥80 bpm; OSA: non-mild: 0/h ≤ AHI < 15/h; moderate: 15/h ≤ AHI < 30/h; severe: 30/h < AHI), and conducted multiple group comparisons of the baPWV values. Only in subjects with Low HR (HR < 70 bpm), significant increase of the baPWV was observed along with an increase in the severity of OSA. OSA obstructive sleep apnea, AHI apnea-hypopnea index, bpm beats per minute, HR heart rate, baPWV brachial-ankle pulse wave velocity; ☨*p* < 0.05 vs. non-mild OSA

We classified the 1611 subjects into three groups based both on the severity of the obstructive sleep apnea (OSA) (AHI: non-mild: 0/h ≤ AHI < 15/h, moderate: 15/h ≤ AHI < 30/h, severe: AHI ≥ 30/h; HR: Low: <70 bpm; Medium: 70 ≤ HR < 80 bpm, High: ≥80 bpm) [15]. As shown in Fig. 3, when the study participants were first classified based on the severity of the OSA, only subjects with non-mild OSA (0/h ≤ AHI < 15/h) showed a significantly increase of the baPWV along with an increase of the HR. No such increase of the HR with an increase of the baPWV was observed in subjects with severe OSA. Similarly, as shown in Fig. 4, when the study participants were classified based on the degree of HR elevation, only in subjects with HRs in the lowest value (<70 bpm) showed a significant increase of the baPWV along with an increase in the severity of OSA, whereas no such increase of the baPWV with increase of the AHI was observed in subjects with HRs in the highest value HR (≥80 bpm).

**Fig. 5:**
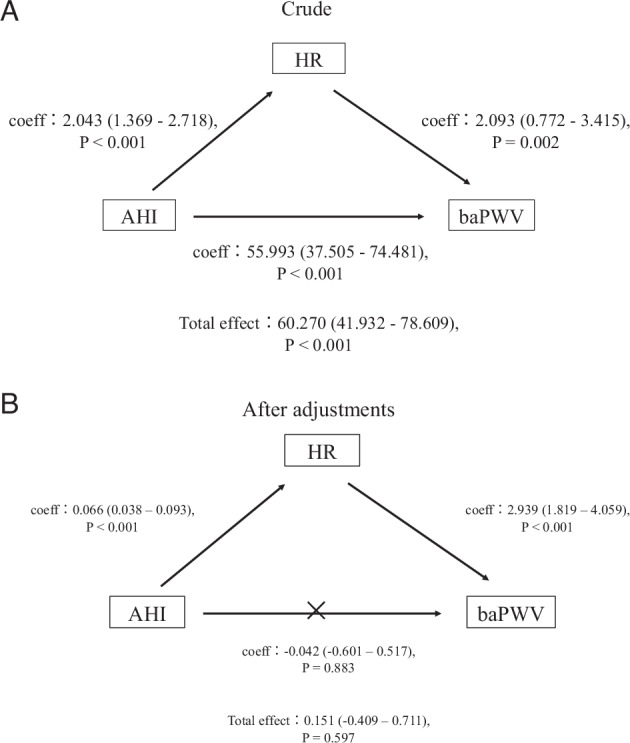
Results of mediation analysis conducted to assess the direct and indirect (via heart rate) associations of the apnea-hypopnea index with the brachial-ankle pulse wave velocity. **A** Crude. In a crude model, the AHI was found to exert both a direct and indirect (via HR) effect on the baPWV. **B** After adjustments. After adjustments for the age, sex, body mass index, mean blood pressure, and medication status, while the AHI was associated with an increase of the baPWV via mediation of the HR, there was no significant direct association between the severity of OSA and the baPWV. OSA obstructive sleep apnea, AHI apnea-hypopnea index, HR heart rate, baPWV brachial-ankle pulse wave velocity

Mediation analysis was conducted to evaluate the direct and indirect relationships between the AHI and PWV mediated by the HR. In a crude model, the AHI was found to exert both a direct and indirect (via HR) effect on the baPWV (Fig. 5A). After adjustments for the age, sex, BMI, MBP, and medication status, the analysis identified AHI as showing a significant association with the PWV mediated by the HR (*P* < 0.001); however, no significant direct relationship was observed between the AHI and PWV (*P* = 0.883) (Fig. 5B). The analysis of the interaction effects on baPWV revealed no significant interaction between HR and AHI (*P* = 0.520) (Supplementary Table 1).

As compared with other markers of the severity of OSA (e.g., arousal index, percentage of slow-wave sleep of total sleep time, percentage of cumulative time with oxygen saturation below 90%, lowest oxygen saturation level), AHI showed a closer association with the baPWV (Supplementary Table 2).

## Discussion

In the present study conducted in a large OSA cohort, mediation analysis demonstrated that while the AHI was directly associated with the HR, its association with the baPWV was mediated via the HR. A recent study has reported that arterial stiffness can vary depending on the time of day, particularly in patients with OSA [16]. However, in the present study, baPWV was measured at around 2:00 PM on the day of hospitalization for polysomnography in all cases, ensuring the validity of the results.

Several previous studies have reported increase of the baPWV values in subjects with OSA, and furthermore, a decrease of the baPWV following initiation of continuous positive airway pressure (CPAP) in OSA patients [17, 18]. Thus, increased arterial stiffness is a robust finding in patients with OSA. Factors implicated in vascular dysfunction associated with OSA, such as inflammation, oxidative stress, hypoxia, and increased blood pressure, have been hypothesized as acting collectively to increase the arterial stiffness [19, 20]. However, the precise mechanisms underlying the increased arterial stiffness in OSA patients have not yet been fully clarified [21].

Existence of an association between elevated HRs and increased values of the baPWV has been reported, and our prospective observational study also reported a significant increase of the PWV with increase of the HR [22]. Two major mechanisms are considered as being involved in the association between HR elevation and increase of the PWV. The first is sympathetic nervous activation, which acts to increase the PWV with simultaneous increase of the HR. Sympathetic nervous activation, in addition to increasing the heart rate, is functionally and structurally involved in increasing the arterial stiffness through mechanisms such as increase of the blood pressure, vascular constriction, and vascular smooth muscle hypertrophy [22, 23]. The second is the direct increase in arterial stiffness mediated by vascular damage due to increased cycle stress on the arterial wall associated with tachycardia itself [24]. However, which of the two mechanisms is more important for the increase in arterial stiffness associated with HR elevation remains under debate.

While an increase of the HR is known in patients with OSA, the relationship between HR and PWV in OSA has not been sufficiently elucidated. We have previously reported OSA as an independent risk factor for PWV progression, but the relationship between the HR and PWV was not adequately examined in the same study [25]. The relationship among AHI, PWV, and HR is complex. As shown in Fig. 2, although the correlations between HR and baPWV, as well as AHI and baPWV, were statistically significant, the strength of these correlations was very modest. Furthermore, in the analysis stratified by OSA severity (Fig. 3), the relationship between HR and baPWV was statistically significant only in patients with non-mild OSA. However, a similar trend was observed in the moderate to severe OSA group, although it was not statistically significant. Likewise, in the HR-stratified analysis, the association between AHI and baPWV was statistically significant only in the Low HR group, but similar trends were observed in the other groups (Fig. 4). Accordingly, to elucidate these associations in greater detail, a mediation analysis was conducted using data from all participants, which revealed the significant association between AHI and PWV mediated by HR. These findings suggested that HR is an important contributing factor to the increase in baPWV among patients with OSA.

### Clinical implications

Recent studies have reported that the decrease in HR observed following initiation of CPAP in OSA patients is important for favorable cardiovascular (CV) outcomes [9, 17]. Therefore, future research is warranted to investigate whether HR elevation in OSA patients serves as a CV risk factor through increasing the PWV. Additionally, it is necessary to examine whether the reduction in PWV through a decrease of the HR following initiation of CPAP might contribute to a reduction in the CV risk. Furthermore, beta-blockers have been reported to be effective to control the blood pressure in patients with OSA [26], and the results of the present study lend support to that finding.

### Study limitations

The present study had some limitations; 1) The Tokyo Sleep Study cohort mostly comprised Japanese men. Thus, the findings of this study need to be confirmed in women and other ethnicities; 2) For the analyses, HRs measured at the time measurement of the baPWV in supine position was used for the analysis. Usually, at the time of blood pressure measurement, HR is measured simultaneously in the sitting position; 3) Antihypertensive medication other than beta-blockers affect the arterial stiffness by mechanisms other than decrease of the HR, and many study participants in the present study were receiving antihypertensive medication. Therefore, medication status was also included as a covariate in the mediation analysis; however, the results remained consistent; 4) Since baPWV is more influenced by blood pressure than other arterial stiffness parameters, such as carotid-femoral PWV or the cardio-ankle vascular index (CAVI). This sensitivity to blood pressure could confound the association between OSA, HR, and arterial stiffness; 5) Although the correlations of baPWV with HR and AHI were statistically significant, the correlation coefficients were very small. Therefore, these relationships warrant further investigation in future studies.

### Perspective of Asia

Although the prevalence of obesity is lower in Asia compared to Western countries, previous reports have indicated that the prevalence of OSA is not substantially different between the two populations [27], likely due to anatomical characteristics of the upper airway specific to Asians. In the present study, the involvement of HR in the mechanism underlying the OSA-related increase in baPWV suggests that the distinct phenotype of OSA observed in non-obese Asian individuals may influence HR.

## Conclusions

In subjects with OSA, increase of the arterial stiffness appears to be mediated by an increase of the HR, and therefore, elevated HR may be one of key to increase arterial stiffness in OSA. Elevated HR per se and/or factors involved in elevating the HR, such as sympathetic nervous activation, may be involved in this increase of the arterial stiffness.

## Supplementary information

Supplement Table 1

Supplement Table 2
