# Supplementary material for: The impact of obstructive sleep apnea and heart rate on arterial stiffness: results from the Tokyo Sleep Heart Study
Source: Hypertens Res. 2025 Aug 22;48(11):2792–800. doi: 10.1038/s41440-025-02334-5 (PMC12586163; doi:10.1038/s41440-025-02334-5)
Supplement: Supplementary file 1 — Supplement Table 1 [file 41440_2025_2334_MOESM1_ESM.docx]

Supplement Table 1.

Multivariate linear regression analyses for baPWV with HR, AHI, and these interaction

|  | baPWV | | |
| --- | --- | --- | --- |
| Variable | β | t | P-value |
| HR | 0.129 | 3.329 | < 0.001 |
| AHI | 0.077 | 0.623 | 0.533 |
| HR×AHI | -0.087 | -0.644 | 0.520 |

After adjustments for the age, sex, body mass index, mean blood pressure, and medication status

Abbreviations: baPWV, brachial-ankle pulse wave velocity; HR, heart rate; AHI, apnea-hypopnea index

HR×AHI, Interaction of HR and AHI
