# Supplementary material for: The impact of obstructive sleep apnea and heart rate on arterial stiffness: results from the Tokyo Sleep Heart Study
Source: Hypertens Res. 2025 Aug 22;48(11):2792–800. doi: 10.1038/s41440-025-02334-5 (PMC12586163; doi:10.1038/s41440-025-02334-5)
Supplement: Supplementary file 2 — Supplement Table 2 [file 41440_2025_2334_MOESM2_ESM.docx]

Supplement Table 2.

The correlation of markers of obstructive sleep apnea severity with baPWV

| variable | r | P-value |
| --- | --- | --- |
| AHI | 0.144 | <0.001 |
| arousal index | 0.112 | <0.001 |
| percentage of slow-wave sleep of total sleep time | -0.125 | <0.001 |
| percentage of cumulative time with oxygen saturation below 90% | 0.039 | 0.121 |
| lowest oxygen saturation level | -0.081 | 0.001 |

Abbreviations: AHI = apnea hypopnea index, baPWV, brachial-ankle pulse wave velocity
